# Chemoradiation for oesophageal cancer: the choice of treatment modality

**DOI:** 10.1186/s13014-023-02290-9

**Published:** 2023-05-31

**Authors:** Pauliina M. Kitti, Maria Faltinova, Juha Kauppi, Jari Räsänen, Tiina Saarto, Tiina Seppälä, Anu M. Anttonen

**Affiliations:** 1 Department of Oncology, HUS Comprehensive Cancer Centre and University of Helsinki, Paciuksenkatu 3, PL 180, 00029 HUS Helsinki, Finland; 2 Department of General Thoracic and Esophageal Surgery, Helsinki University Hospital and University of Helsinki, Helsinki, Finland

**Keywords:** Oesophageal cancer, Chemoradiotherapy, Neoadjuvant therapy, Surgical oncology, Radiotherapy, Dosimetric parameters, Treatment modality

## Abstract

**Background:**

Locally advanced oesophageal cancer can be treated with definitive chemoradiation (dCRT) or with neoadjuvant chemoradiation followed by surgery (nCRT + S), but treatment modality choice is not always clear. The aim of this study was to investigate the factors associated with the choice of treatment modality in locally advanced oesophageal cancer.

**Methods:**

This was a retrospective cohort study of 149 patients treated with dCRT(n = 85) or nCRT + S (n = 64) for oesophageal cancer in Helsinki University Hospital in 2008–2018. Logistic regression was used to analyse factors associated with choice of treatment modality and to compare dosimetric factors with postoperative complications. Multivariate analyses identified factors associated with survival.

**Results:**

Surgery was performed after chemoradiation as planned on 64/91 patients (70%). 28/64 had pathological complete response (44%). Probability of nCRT + S was higher in stages I-III versus IV (OR 3.62, 95% CI 1.53–8.53; *P* = .003), ECOG 0–1 versus 2 (OR 6.99, 95% CI 1.81–26.96; *P* = .005) or in the middle/lower vs upper oesophageal tumours (OR 5.61, 95% CI 1.83–17.16, *P* = .003). Probability for surgery was lower, if patient had lost > 10% of body weight (OR 0.46, 95% CI 0.21–0.98, *P* = 0.043). Patients in the nCRT + S group had significantly better median overall survival (mOS) and local control than the dCRT group (60 vs. 10 months, *P* < .001 and 53 vs. 6 months, *P* < 0.0001, respectively). 10/85 (12%) patients died within three months after dCRT. In multivariate analysis, nCRT + S was associated with improved mOS (HR 0.28, 95% CI 0.17–0.44, *P* < .001). Current smokers had worse mOS (HR 2.02, 95% CI 1.04–3.92, *P* = .037) compared to never-smokers. No significant dosimetric factor associated with postoperative complications was found.

**Conclusion:**

The overall clinical status of the patients and the stage of the cancer guide the choice of treatment modalities, leading to overtreatment. Patients with better prognoses were more likely operated after chemoradiation, although there is no evidence of OS benefit in previous randomized trials. On the other hand, the prognosis was poor for patients with poor general health and advanced cancers, despite the chemoradiation. Thus, there are signs of overtreatment. MDT practice should be recommended to optimise the choice of treatment modalities. Smoking status is an independent factor associated with survival.

## Introduction

Locally advanced oesophageal cancer can be treated with neoadjuvant chemoradiation followed by surgery (nCRT + S) or with definitive chemoradiation (dCRT) [1]. A recent meta-analysis [2] revealed that the overall survival (OS) of patients treated with nCRT + S was significantly better than the OS of patients treated with dCRT. However, the OS effect of the surgery has been challenged in two randomized studies [3, 4] that compared dCRT and nCRT + S and revealed a similar OS despite surgery, but these studies included mostly squamous cell carcinomas and less adenocarcinomas. The probability for surgery is higher e.g. in stages II (vs III) and tumours of the lower oesophagus; the probability is lower in older patients and in those with multiple comorbidities [5].

Chemoradiation (CRT) may lead to many complications, including pulmonary and cardiac complications [6]. Higher doses of radiation to lungs and heart may increase radiotherapy side effects and postoperative complications [7–10].

The aim of this study was to report the outcomes after dCRT or nCRT + S and to identify factors associated with choice of treatment modality and OS. In addition, we aimed to determine if dosimetric parameters are associated with post-surgical pulmonary and cardiac complications after nCRT + S.

## Materials and methods

### Study cohort

Medical records of patients with oesophageal cancer treated with dCRT or nCRT + S between 2008 and 2018 in the Comprehensive Cancer Center at Helsinki University Hospital, Finland, were retrospectively analysed with the approval of our institutional review board. A total of 174 patients were identified. Twenty-five patients were excluded from the study (6 received chemoradiation for residual cancer or metastasis, 12 did not complete radiation, 2 received chemoradiotherapy simultaneously also for head and neck cancer, 4 received chemoradiation postoperatively, and 1 patient moved to another area). The final study cohort consisted of 149 patients. The cohort was analysed in September 2021. Date of diagnosis was defined as the date of biopsy-proven diagnosis.

All patients were staged with CT and most (99%, n = 147) with 18-FDG-PET/CT. Endoscopic ultrasound was used for staging for 46% (n = 69) and laparoscopy for 1% (n = 2). Staging was performed according to the American Joint Committee on Cancer 8^th^ edition. This staging indicated that 6 patients with supraclavicular lymph-node metastasis were stage IVb (but were included in this study) and 11 patients were stage IVb due non-regional lymph-node metastasis and these non-regional lymph-node metastasis were included in the radiation field. No patient had visceral or bone metastasis.

Half of the patients (53%, n = 79) lost > 10% body weight before diagnosis. Most patients (83%, n = 124) visited a nutritional therapist. A PEG tube was inserted for 33 patients (22%). An oesophageal stent was placed in 58 patients (39%) before CRT.

### Treatment

CRT consisted of platinum-based chemotherapy delivered concurrently with radiation therapy; 109 patients (73%) received paclitaxel-carboplatin, 22 patients (15%) cisplatin-fluorouracil, 16 patients (11%) weekly cisplatin, and 2 patients (1.3%) cisplatin-etoposide. Elective nodal irradiation was included based on the decision of the radiation oncologist. For motion control, 4D-CT was used for 25.5% of patients (n = 38). Four patients underwent chemotherapy before CRT; these patients had adenocarcinomas that were unsuitable for surgery after chemotherapy. Twelve patients had gaps in radiotherapy but completed treatment. Forty-nine patients (32.9%) did not complete concurrent chemotherapy due to infections (21 patients), haematological toxicity (14 patients), and deterioration of overall clinical status (4 patients). Patients were assigned to dCRT or nCRT + S according to physician and patient preferences. Dose-volume histogram (DVH) data were extracted from Eclipse treatment planning systems (Varian, Palo Alto, USA). The following dosimetric parameters were computed using either Pencil Beam Convolution or Anisotropic Analytical Algorithms: mean heart dose, heart V30, mean lung dose, and lung V5 and V20.

### Statistical analysis

Descriptive statistics, including medians, interquartile ranges, means, ranges, frequencies, and percentages were used for patient characteristics. Comparisons between treatments were performed using a one-way ANOVA for continuous variables and Fisher’s exact test and χ^2^ for categorical variables. If variances in groups were not equal, analysis was performed using the Kruskal–Wallis test. The primary endpoint for all patients was OS, which was calculated from the completion of CRT to the time of death or the time of the final cohort analyses in September 2021. The Kaplan–Meier method was used to estimate survival and local recurrence; significance was determined with the log-rank test. Multivariate analyses were performed with the Cox proportional hazards model. Binary logistic regression was used to compare DVH factors with postoperative complications and mortality and to compare stage, Eastern Cooperative Oncology Group (ECOG), location of primary tumour, weight loss, age and gender with choice of treatment modality. IBM SPSS Statistics version 25 was used for data analysis. Statistical significance was set at *P* < 0.05.

## Results

Patient characteristics are shown in Table 1. Most (84.6%) patients were ECOG performance status 0–1. The predominant histological type was squamous cell carcinoma (84.6%). The groups (nCRT + S vs dCRT) had differences in stage, tumour location, and ECOG. Median age was 65 years, 13 patients (8.7%) were over the age of 75.

**Table 1:** Patient characteristics

	All n	definitive chemoradiation n	neoadjuvant chemoradiation and surgery n	*p*
	149 (100%)	85 (57.0%)	64 (43.0%)	
Gender				0.732
Male	94 (63.1%)	55 (64.7%)	39 (60.9%)	
Female	55 (36.9%)	30 (35.3%)	25 (39.1%)	
Median age at diagnosis (range)	65 (39–80)	64 (47–78)	65 (39–80)	0.372
ECOG performance status				< 0.005
0	24 (16.1%)	6 (7.1%)	18 (28.1%)	
1	102 (68.5%)	59 (69.4%)	43 (67.2%)	
2	23 (15.4%)	20 (23.5%)	3 (4.7%)	
Smoking				0.186
Never	24 (16.1%)	9 (10.6%)	15 (23.4%)	
Current smoker	77 (51.7%)	48 (56.5%)	29 (45.3%)	
Ex-smoker	37 (24.8%)	21 (24.7%)	16 (25.0%)	
Unknown	11 (7.4%)	7 (8.2%)	4 (6.3%)	
BMI median (range)	22.8 (13.3–42.6)	22.9 (13.3–42.6)	22.8 (16.6–34.4)	0.445
Histology				0.665
Squamous cell carcinoma	126 (84.6%)	69 (81.2%)	57 (89.1%)	
Adenocarcinoma	13 (8.7%)	9 (10.6%)	4 (6.3%)	
Other	10 (6.7%)	7 (8.2%)	3 (4.7%)	
Lesion location				0.005
Upper	30 (20.1%)	25 (29.4%)	5 (7.8%)	
Middle	70 (47.0%)	35 (41.2%)	35 (54.7%)	
Lower	49 (32.9%)	25 (29.4%)	24 (37.5%)	
Stage 8th AJCC				0.002
I	2 (1.3%)	1 (1.2%)	1 (1.6%)	
II	35 (23.5%)	14 (16.5%)	21 (32.8%)	
III	60 (40.3%)	29 (34.1%)	31 (48.4%)	
IVa	35 (23.5%)	26 (30.6%)	9 (14.1%)	
IVb	17 (11.4%)	15 (17.6%)	2 (3.1%)	
Median dose, Gy (range)	50.4(41.4–70)	50.4 (41.4–70)	45 (41.4–66)	< 0.0001
Radiotherapy modality				0.116
3D CRT	22 (14.8%)	14 (16.5%)	8 (12.5%)	
IMRT	56 (37.6%)	37 (43.5%)	19 (29.7%)	
VMAT	70 (47%)	34 (40.0%)	36 (56.3%)	
PTV cm^3^, median (range)	554.8 (121.4–1821.3)	639.2 (199.1–1490.5)	467.2 (121.4–1821.3)	0.009
PTV cm, median (range)	16.6 (8.6–31.4)	17.6 (8.7–29.9)	15.5 (8.6–31.4)	0.033
Late toxicity after chemoradiation				
Pneumonitis				0.238
Grade 1–2	14 (9.4%)	10 (11.8%)	4 (6.3%)	
Grade 3–4	4 (2.7%)	1 (1.2%)	3 (4.7%)	
Oesophageal stricture, any grade	28 (18.8%)	13 (15.3%)	15 (23.4%)	0.063
Fistula, any grade	27 (18.1%)	16 (18.8%)	11 (17.2%)	0.76

ECOG: Eastern Cooperative Oncology Group; BMI: Body Mass Index; AJCC: American Joint Committee on Cancer; Gy: Gray; 3D CRT: Three-dimensional conformal radiation therapy; IMRT: intensity-modulated radiotherapy; VMAT: Volumetric modulated arc therapy; PTV: planning target volume

Although surgery was considered for 91 patients, 17 had metastatic cancer based on preoperative images, 9 were not physically eligible for surgery, and 1 patient died before surgery. Radical surgery was performed for 64 patients (42.7% of all patients), of which 41 patients (64.1%) had minimally invasive esophagectomy and 23 patients (35.9%) had open esophagectomy. Surgery was performed median 90 days (range 50–218) after completion of CRT. Fifty-eight patients (92.1%) had clear margins in surgery. Pathological complete response after nCRT was achieved in 28 patients (43.8%). Eighty-five patients (57.0%) were treated with dCRT, but 9 of these patients received only preoperative dose (41.4 Gy), because they were planned to have surgery, but were not, due to preoperatively found metastatic disease or poor general condition.

One-hundred-and-three patients (69.1%) received elective nodal irradiation (ENI). Twenty-five (83.3%) of patients with upper oesophageal tumour received ENI, compared to 78 patients (65.5%) with lower or middle oesophageal tumours (*p* = 0.059). The volumes of PTV were larger with ENI than without ENI (median 621cm^3^ vs. 349 cm^3^, *p* < 0.0001). Mean lung dose (MLD) was higher, if elective lymph nodes were irradiated vs not (10.8 Gy vs 9.4 Gy, *P* = 0.037) and with greater T stage (T1-2 7.9 Gy vs. T3-4 11.2 Gy, *P* < 0.0001).

The most common acute side effect of CRT was esophagitis (any grade n = 93 [62.4%], grade ≥ 3 n = 12 [8.1%]) followed by fatigue (n = 55 [36.9%] grade ≥ 3 n = 7 [4.7%]), haematological toxicity (n = 41 [27.5%], grade ≥ 3 n = 14 [9.4%]), nausea (n = 33 [22.1%] grade ≥ 3 n = 1 (0.7%)), and weight loss (n = 28 [18.8%], grade ≥ 3 n = 4 [2.7%]). No significant difference in late toxicities was found between the treatment modalities (Table 1).

Postoperative complications are shown in Table 2. The most common pulmonary complications were pneumonia and pleural effusion, and the most common gastrointestinal complications were anastomosis leakage (n = 6) and tracheal fistula (n = 2). No significant DVH factor that explained postoperative pulmonary and cardiac complications, or 90-day mortality were found in logistic regression analysis (Table 3).

**Table 2:** Postoperative complications and mortality

Event	Any grade, n (%)	Grade ≥ 3, n (%)
Postoperative complications (n = 64)		
Pulmonary	31 (48.4)	15 (23.4)
Cardiac	9 (14.1)	5 (7.8)
Chylothorax	7 (10.9)	
Gastrointestinal	18 (28.1)	13 (20.3)

Postoperative mortality (n = 64)	n (%)
7 days	0 (0)
8–30 days	1 (1.6)
31–60 days	1 (1.6)
62–90 days	2 (3.1)

**Table 3:** Univariate binary logistic regression analysis of dosimetric factors and postoperative complications and mortality

Characteristics	Pulmonary complications		Cardiac complications		90-day mortality	
	OR (95% CI)	*p*-value	OR (95% CI)	*p*-value	OR (95% CI)	*p*-value
PTV volume	1.00 (1.00–1.00)	0.911	1.0. (1.00–1.00)	0.960	1.00 (1.00–1.01)	0.100
Lung V5%	1.01 (0.93–1.10)	0.864	0.96 (0.89–1.05)	0.342	1.13 (0.91–1.41)	0.265
Lung V20%	1.11 (0.92–1.34)	0.261	1.05 (0.86–1.28)	0.653	1.10 (0.70–1.71)	0.685
Mean lung dose	0.68 (0.33–1.41)	0.300	1.21 (0.57–2.55)	0.626	0.43 (0.62–2.98)	0.391
Heart V30%	0.97 (0.92–1.05	0.665	.96 (0.87–1.05)	0.362	1.00 (0.83–1.19)	0.965
Mean heart dose	1.06 (0.91–1.23)	0.488	1.07 (0.88–1.31)	0.483	0.93 (o.64–1.36)	0.709

PTV: Planning target volume; V5, V20, and V30, percentage volumes receiving specific doses of 5, 20 and 30 Gy

Probability of nCRT + S was higher in stages I-III vs IV (odds ratio [OR] 3.62, 95% confidence interval [CI] 1.53–8.53; *P* = 0.003) or ECOG 0–1 vs. 2 (OR 6.99, 95% CI 1.81–26.96; *P* = 0.005) or if the tumour was in the middle or lower oesophagus vs upper (OR 5.61, 95%CI 1.83–17.16, *P* = 0.003). Probability for surgery was lower, if patient had lost > 10% of body weight at the time of the diagnosis (OR 0.46, 95% CI 0.21–0.98, *P* = 0.043). Gender (OR 1.12, 95% CI 0.51–2.46, *P* = 0.769) or age (OR 1.00, 95% CI 0.95–1.05, *P* = 0.970) did not have effect on the treatment modality.

**Fig. 1:**
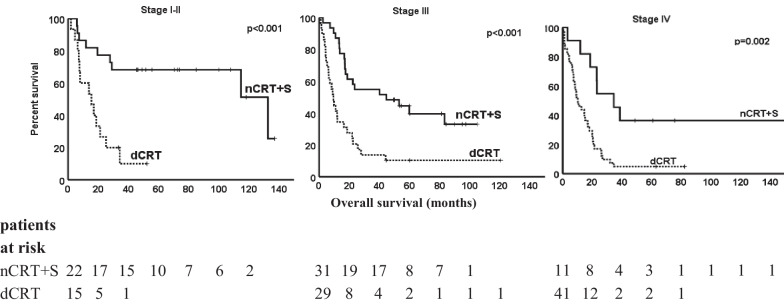
Comparisons stage by stage of overall survival with definitive chemoradiation and neoadjuvant chemoradiation and surgery. dCRT: definitive chemoradiation; nCRT + S: neoadjuvant chemoradiation and surgery

The median follow up was 66.7 months (range 33.3–154.4) for surviving patients. For all patients, median overall survival (mOS) after completion of CRT was 18 months (range 0.6–154.4). Five-year survival rate was 16.8%. Stage-by-stage comparisons of the groups are presented in Fig. 1. Of all the patients, mOS of stage IVa patients was16.9 months and 14.9 months for IVb patients (*p* = 0.008). Median OS of patients with M1 disease with lymph node metastasis in supraclavicular area was 14.7 months, and 9.4 months in M1 patients with other non-regional lymph node metastasis (*p* = 0.087).

Patients who underwent nCRT + S had significantly better mOS (60 vs. 10 months, *P* < 0.001) than those who underwent dCRT. Median OS was not reached for patients with pCR after surgery. Nineteen of them (68%) were still alive in the end of follow-up. Ten patients (11.8%) died within 3 months after completion of dCRT. Altogether 21 (24.7%) patients died within 6 months.

Median local recurrence free survival was 6.5 months after dCRT (95% CI 4.86–8.22) and 53.2 months after nCRT and surgery (95% CI 0.00–107.74) *p* < 0.0001.

In univariate analysis, factors associated with better OS were non-smoking status, lower ECOG and stage, completion of chemotherapy, surgery, smaller PTV volume, and lower mean lung dose (Table 4). In multivariate Cox regression analysis, surgery and non-smoking status were statistically significantly associated with improved OS (Table 4). Current smokers had a 2.02-fold risk of death compared to never-smokers.

**Table 4:** Univariate and multivariate analyses of the association of clinical and treatment-related factors with overall survival

Factor	No. of patients (%)	Univariate analysis		Multivariate analysis	
		Median OS in months (95% CI)	*P* value	Hazard ratio (95% CI)	*P* value
Age in years			0.395		
Median 64,9					
<65	75 (50.3)	17 (14–20)			
≥ 65	74 (49.7)	20 (14–26)			
Sex			0.895		
Female	55 (36.9)	18 (11–25)			
Male	94 (63.1)	17 (13–22)			
Smoking status			**0.018**		
Nonsmoker	24 (16.1)	60 (NA)		Ref.	
Former smoker	37 (24.8)	18 (12–25)		1.58 (0.75–3.34)	0.228
Current smoker	77 (51.7)	14 (9–19)		2.02 (1.04–3.92)	**0.037**
Unknown	11 (7.4)	17 (3–31)		1.62 (0.60–4.34	0.339
ECOG performance status			**0.015**		
0	24 (16.1)	20 (6–35)		Ref.	
1	102 (68.5)	19 (14–23)		0.75 (0.41–1.38)	0.356
2	23 (15.4)	11 (8–13)		0.74 (0.33–1.65)	0.460
Stage			**0.008**		
I–II	37 (24.8)	29 (0–96)		Ref	
III	60 (40.3)	17 (14–20)		1.29 (0.69–2.41)	0.423
IVa	35 (23.5)	17 (6–28)		1.17 (0.61–2.25)	0.629
IVb	17 (11.4)	15 (7–22)		1.36 (0.63–2.96)	0.437
Histology			0.874		
Squamous cell carcinoma	126 (84.6)	17 (13–21)			
Other	23 (15.4)	20 (15–27)			
BMI			0.164		
Median 22.8 (range 13.3–42.6)					
<20	34 (22.8)	9 (0–18)			
20–24.99	65 (43.6)	18 (15–21)			
>25	48 (32.9)	23 (6–40)			
Weight loss >10% before chemoradiation			0.136		
Yes	79 (53.0)	13 (9–18)			
No	70 (47.0)	21 (17–25)			
Completion of chemotherapy			**0.028**		
Yes	100 (67.1)	19 (16–23)			
No	49 (32.9)	12 (7–18)		0.70 (0.45–1.09)	0.116
Surgery			**<0.0001**		
Yes	64 (43.0)	60 (12–108)		**0.28 (0.17–0.44)**	**<0.0001**
No	85 (57.0)	10 (7–13)			
Radiation modality			0.531		
3D CRT	22 (14.8)	19 (6–31)			
IMRT	56 (37.6)	17 (15–19)			
VMAT	70 (47.0)	17 (8–26)			
Radiation dose, Gy			0.422		
41.4	30 (20.1)	23 (16–30)			
45	39 (26.2)	19 (5–33)			
50–50.4	49 (32.9)	17 (13–21)			
54–60	23 (15.4)	15 (2–28)			
61–70	8 (5.4)	26 (13–39)			
PTV, cm^3^			**0.038**		
Median 555 (range 121–1821)					
<555	75 (50.3)	22 (18–27)		Ref.	
≥555	74 (49.7)	14 (7–20)		1.00 (0.99–1.00)	0.456
Mean lung dose, Gy			**0.040**		
Median 10,1 (range 4,0–21,8)					
<10	71 (49.0)	23 (18–28)		Ref.	
≥10	74 (51.0)	12 (7–17)		1.05 (0.99–1.12)	0.116
Lung V5, %			0.182		
Median 55.6 (range 12.0-98.4)					
<55	71 (49.0)	21 (17–26)			
≥55	74 (51.0)	14 (7–20)			
Lung V20, %			0.052		
Median 14.6 (range 2.2-49.1)					
<15	77 (53.1)	23 (17–29)			
≥15	68 (46.9)	13 (7–20)			
Mean heart dose, Gy			0.922		
Median 17.9 (range 0.2-37.5)					
<18	72 (50.0)	19 (15–23)			
≥18	72 (50.0)	15 (8–21)			
Heart V30, %			0.607		
Median 16.5 (range 0.0–85.9)					
<17	73 (50.3)	20 (15–25)			
≥17	72 (49.7)	14 (7–21)			
Local recurrence			**<0.0001**		
Yes	59	15 (10–20)		0.74 (0.48–1.14)	0.722
No	90	23 (7–38)		Ref.	

ECOG: Eastern Cooperative Oncology Group; BMI: Body Mass Index; Gy: Gray; 3D CRT: Three-dimensional conformal radiation therapy; IMRT: intensity-modulated radiotherapy; NA: not applicable; PTV: planning target volume; V5, V20, and V30, percentage volumes receiving specific doses of 5, 20 and 30 Gy; VMAT: Volumetric modulated arc therapy. Bold values denote statistical significance at the p < 0.05 level

## Discussion

We aimed to identify factors associated with the choice of treatment modality in the treatment of locally advanced oesophageal cancer. The overall clinical status of the patients and the stage of cancer guide the choice of treatment modalities, leading to overtreatment.

General condition of the patient, the extent of the disease, the location of the tumour in the oesophagus, weight loss, but not age or gender, were associated with the choice of the treatment modality. Thus, patients with better prognoses were more likely to be operated after chemoradiation.

In the present study population, surgery was performed on 42.7% of patients after CRT, which is consistent with the previously reported 27–58% [5, 12, 14]. Surgery was planned for 61% of the patients, but due to advanced disease or poor performance status, not implemented for all of those. After nCRT + S, pCR was achieved in 28 patients (44%). The mOS of 60 months after nCRT + S in the present study was better than in most previously reported studies, where mOS has been 16–62 months [3, 4, 11, 12]. For patients with pCR mOS was yet not reached, raising the question whether surgery was needed for all cases. However, as stated in a recent review [17], these complete responders are difficult to identify before surgery.

In two previous randomized studies [3, 4] of mostly squamous cell carcinomas patients like in the present study, no superiority of nCRT + S over dCRT in OS was found, but [3, 4] the local recurrence rate was lower after nCRT + S. In our study, both mOS and median local recurrence free survival were better after surgery, apparently due to selection of the patients. It seems that surgeries were performed on all eligible patients, even though according to the randomized studies [3, 4], these patients did not necessarily benefit from surgery. In contrast, mOS after CRT was poorer in our study (10 months) when compared with the previously reported 11–28 months [3, 4, 11–13]. Our population differed from the previous ones, that we also included stage IVb patients. In addition, in our cohort some patients received only the preoperative dose of 41.4 Gy, which is below the recommended radiation dose in definitive setting [1], because the planned surgery was nor performed. This highlights the importance of initial clinical evaluation, to avoid too low radiation doses, when the probability of surgery is minimal. However, considering the fact that 10 patients (12%) died within 3 months after completion of CRT, patient selection for CRT was likely not optimal. It is likely that the patients were too fragile, and the cancers were too advanced. The rather large percentage of chemotherapy interruptions (33% in the whole cohort) and the 12 patients who were excluded from this study after interrupting radiotherapy may also indicate that some patients were overtreated.

At the time of the study, multidisciplinary teams (MDT) were not involved in the decision making. MDT are essential part of treatment, especially in gastroesophageal cancers, as stated in recent ECCO guideline [16]. The purpose of MDT is to evaluate treatment plans multidisciplinary together with oncologists, radiation oncologists and surgeons to optimise the treatment. Extended MDT with geriatricians and palliative specialists is needed to plan treatment for patients with poor performance status and advanced disease to avoid overtreatment.

In multivariate analysis, the only other significant association with mOS besides surgery was current smoking, which increased the risk of death by 2.02-fold compared to never-smokers. The impact of smoking on the poorer prognosis of patients treated with CRT has also been shown in a previous study [15]. This emphasizes the importance of smoking cessation.

Lung radiation dose was associated with survival outcomes in a previous study[10]. In our study although patients with smaller lung and heart radiation doses seemed to have better mOS, the differences were not statistically significant. The positive association between lung dose and T stage could at least partly explain this difference.

After surgery, almost half (48%) of the patients had pulmonary complications (e.g., pneumonia or pleural effusion) and 28% had gastrointestinal complications (e.g., anastomosis leakage, ileus). This is consistent with previous studies (pulmonary complications in 25–63% and gastrointestinal complications in 22–23%) [2, 8, 9, 18]. We did not find a correlation between complications and dosimetric factors. Previous studies suggested a correlation between lung radiation dose and postoperative pulmonary complications and heart radiation dose and cardiac complications [8, 10, 19]. Despite these reported complications, postoperative mortality remained low.

There are some limitations of this study. This includes the retrospective design and therefore the lack of more detailed information of the decision making. Of note, for the generalizability of our finding, our cohort consists of only a few patients with adenocarcinoma, based on the clinical practice of treatment of adenocarcinoma with neoadjuvant chemotherapy instead of chemoradiation. Propensity score matching was not possible due to patient heterogeneity and the small number of patients. The strength of this study is its population-based real-life data that demonstrate how treatment modalities are chosen outside of the RCTs.

## Conclusions

The overall clinical status of the patients and the stage of the cancer guided the choice of treatment modalities leading to overtreatment. Patients with better prognoses were more likely to be operated after chemoradiation, although there is no evidence of OS benefit in previous randomized trials. On the other hand, the prognosis was poor for patients with poor general health and advanced cancers despite of the chemoradiation. MDT practice should be recommended to optimise the choice of treatment modalities. Of note, smoking status is an independent factor associated with survival.

## Data Availability

Due to the nature of this research, the study participants did not agree for their data to be shared publicly, thus supporting data are not available.

## Abbreviations

18-FDG-PET/CT: 8F-fluorodeoxyglucose positron emission tomography/computerized tomography
3D CRT: Three-dimensional conformal radiation therapy
AJCC: American joint committee on cancer
ANOVA: Analysis of variance
BMI: Body mass index
CI: Confidence interval
CT: Computed tomography
dCRT: Definitive chemoradiation
DVH: Dose-volume histogram
ECOG: Eastern cooperative oncology group
Gy: Gray
HR: Hazard ratio
SPSS: Statistical package for the social sciences
IMRT: Intensity-modulated radiotherapy
MDT: Multidisciplinary team
nCRT + S: Neoadjuvant chemoradiation and surgery
OR: Odds ratio
OS: Overall survival
PEG: Percutaneous endoscopic gastrostomy
PTV: Planning target volume
V5, V20, and V30: Percentage volumes receiving specific doses of 5, 20 and 30 Gy
VMAT: Volumetric modulated arc therapy
